# Altered metabolic connectivity between the amygdala and default mode network is related to pain perception in patients with cancer

**DOI:** 10.1038/s41598-022-18430-2

**Published:** 2022-08-18

**Authors:** Wen-Ying Lin, Jen-Chuen Hsieh, Ching-Chu Lu, Yumie Ono

**Affiliations:** 1 Department of Anesthesiology, National Taiwan University Cancer Center, Taipei, Taiwan; 2 Department of Anesthesiology, National Taiwan University Hospital, Taipei, Taiwan; 3 Department of Biological Science and Technology, College of Biological Science and Technology, National Yang Ming Chiao Tung University, Hsinchu, Taiwan; 4 Department of Nuclear Medicine, National Taiwan University Hospital, Taipei, Taiwan; 5 School of Science and Technology, Meiji University, Kawasaki, Japan

**Keywords:** Cognitive neuroscience, Emotion, Sensory processing, Head and neck cancer

## Abstract

We investigated the neural correlates for chronic cancer pain conditions by retrospectively analyzing whole brain regions on 18F-fluoro-2-deoxyglucose-positron emission tomography images acquired from 80 patients with head and neck squamous cell carcinoma and esophageal cancer. The patients were divided into three groups according to perceived pain severity and type of analgesic treatment, namely patients not under analgesic treatment because of no or minor pain, patients with good pain control under analgesic treatment, and patients with poor pain control despite analgesic treatment. Uncontrollable cancer pain enhanced the activity of the hippocampus, amygdala, inferior temporal gyrus, and temporal pole. Metabolic connectivity analysis further showed that amygdala co-activation with the hippocampus was reduced in the group with poor pain control and preserved in the groups with no or minor pain and good pain control. The increased although imbalanced activity of the medial temporal regions may represent poor pain control in patients with cancer. The number of patients who used anxiolytics was higher in the group with poor pain control, whereas the usage rates were comparable between the other two groups. Therefore, further studies should investigate the relationship between psychological conditions and pain in patients with cancer and analyze the resultant brain activity.

*Trial registration*: This study was registered at clinicaltrials.gov on 9/3/20 (NCT04537845).

## Introduction

Pain is one of the most prevalent symptoms in patients with cancer. Overall, 55% of patients with cancer experience pain during treatment, and the prevalence increases up to 80% depending on the stages or sites of the cancer^1–3^. The actual etiology of cancer pain remains poorly understood owing to the various potential direct and indirect causes^4^, which has resulted in approximately one-fourth to half of the patients with cancer reporting cancer-pain undertreatment^5–7^.

Evidence suggests a strong interaction between pain and emotion, that is, negative emotions can worsen pain experiences^8^, and chronic pain also frequently coexists with anxiety and depression^9^. The limbic system integrates information from the sensory and affective components of pain and anxiety, which include the limbic lobe, hippocampal formation, amygdala, septal area, and hypothalamus^10,11^. The altered response of the amygdala to chronic pain may further affect the functional brain networks related to interoception, emotion, and cognitive status, all of which may result in maladaptive pain perception^12,13^. Elucidating the cortical representation of cancer pain perception and the differences in cortical responses between well-managed and poorly-managed chronic cancer pain could further improve our understanding of the central mechanism of chronic cancer pain and lead to potential treatments.

Therefore, we investigated the resting-state brain activity of patients with cancer reporting various intensities of perceived pain by analyzing 18F-fluoro-2-deoxyglucose (FDG)-positron emission tomography/computed tomography (PET/CT) images acquired for routine clinical examination purposes. FDG-PET images can be used for quantitative evaluations in clinical oncology^14^ and serve as a functional neuroimaging tool to investigate brain areas and metabolic connectivity in various pain conditions^15–18^. Our previous animal study on cancer-induced bone pain confirmed the reliability of FDG-PET for cancer pain investigations^19^. However, the utilization of FDG-PET in patients with cancer pain had been limited to a small-group study^20^ with results that were difficult to interpret because of the various types of cancers and other clinical conditions included in the sample.

We investigated signature cortical responses reflecting good or poor pain control by comparing the cortical activity of 80 patients with cancer across three groups of perceived pain intensities and on pain control statuses based on analgesic use. We hypothesized that cancer pain activates the amygdala and limbic system compontents involved in pain-emotion interactions and that this activity is inhibited in patients who are appropriately treated with analgesics. We also investigated differences in the cortical metabolic network emerging from these medial temporal regions within other resting-state networks to determine the functional organization in conditions of good and poor control of chronic cancer pain. The working hypothesis is that the pain-related changes in the metabolic network may also be ameliorated under the good pain control conditions.

## Methods

### Study design

Patients who underwent FDG-PET/CT between March 2015 and July 2020 at the National Taiwan University Hospital were retrospectively enrolled. This study was approved by the institutional ethics committees of the National Taiwan University Hospital and conducted in accordance with the Declaration of Helsinki. The requirement for informed patient consent was waived owing to the retrospective nature of the study. We included patients diagnosed with head and neck squamous cell carcinoma or esophageal cancer who completed a PET/CT study for the clinical indication of cancer staging. These cancer types were selected because they share a cancer cell origin, clinical risk factors, and genetic alterations in tumors^21–24^. Patients with brain metastases based on brain magnetic resonance imaging or CT and patients without a pain rating on the day of the PET scan were excluded. Data collected for analysis included age, sex, cancer diagnosis, pain condition including location, pain score, medication, and FDG-PET/CT findings of the patients.

We divided the patients into three groups: patients treated with analgesics with good pain control (AG group, numerical rating scale [NRS] scores ranging from 0 [no pain] to 10 [worst pain imaginable]; < 3, range 0–2), patients treated with analgesics with poor pain control (AP group, NRS ≥ 3, range 3–8), and patients with no or ignorable pain and not receiving analgesic treatment (N group, NRS = 0 or 1) (Table 1). The NRS represents the highest pain rating on the day of the PET scan. Analgesics were divided into three types: non-opioids (acetaminophen, non-steroidal anti-inflammatory drugs, and oxcarbazepine), weak opioids (tramadol, codeine, and nalbuphine), and strong opioids (morphine, fentanyl, and oxycodone). Anxiolytic agents included benzodiazepines, zopiclone, zolpidem, mirtazapine, quetiapine, and flupentixol/melitracen. The threshold of NRS ≥ 3 was selected to divide the AP and AG groups based on an analgesic-prescription criterion used in practice. In total, 27, 24, and 29 patients (n = 80) were included in the N, AG, and AP groups, respectively.

**Table 1:** Patient demographics.

	No analgesic use (n = 27)	Analgesic use with good pain control (n = 24)	Analgesic use with poor pain control (n = 29)	*p* value
Age, years	54.5 (12.21)	55.3 (10.85)	56.0 (9.95)	0.887
Male, n (%)	23 (85.1)	21 (87.5)	25 (86.2)	0.971
Head-neck cancer, n	19*	21^#^	25^#^	
Esophageal cancer, n	10*	4^#^	5^#^	
Pain score, NRS	0.14 (0.36)	0.83 (0.86)	3.72 (1.38)	**< 0.001**
Cancer recurrence, n	3	6	13	**0.017**
Stage, Tumor	3.00 (0.97)	2.88 (1.07)	3.12 (1.02)	0.799
Node	1.75 (1.03)	1.94 (0.87)	2.25 (0.68)	0.235
Duration of opioid use, days		23.7 (22.69)	23.92 (35.11)	0.985
Analgesics				**< 0.001**
Non-opioids, n		14	3	
Weak opioids, n		8	14	
Strong opioids, n		2	12	
Anxiolytic use, n	2	3	13	**0.0014**

Values are presented as mean (standard deviation). Bold values represent statistical significance.

*NRS* numeric rating scale.

*Two patients had both cancers.

^#^One patient had both cancers.

### FDG-PET image acquisition and analysis

PET/CT image acquisition followed the routine protocol of the National Taiwan University Hospital PET Imaging Center. All patients fasted for at least 6 h, and their blood glucose levels were measured to be below 120 mg/dL before receiving intravenous 5–6 MBq/kg of FDG. Whole-body scanning from the skull to the proximal thigh started 45 min after the FDG injection using a PET/CT scanner (GE DISCOVERY ST; GE Medical Systems, Milwaukee, WI; matrix size = 192 × 192, field of view = 300 mm, voxel size = 1.56 × 1.56 × 3.27) or Biograph mCT (Siemens Healthcare, Erlangen, Germany; matrix size = 200 × 200, field of view = 500 mm, voxel size = 2.5 × 2.5 × 3).

PET image analysis was performed using MATLAB (version 2019b, MathWorks, Natick, MA, USA) and Statistical Parametric Mapping (SPM12; Wellcome Department of Cognitive Neurology, London, UK). We extracted the head region from the raw PET scans for each patient from the original Digital Imaging and Communications in Medicine files and reconstructed the three-dimensional images of cerebral FDG uptake. The individual images were co-registered and normalized to the standard stereotactic space in reference to a standardized FDG-PET template (2 × 2 × 2 mm voxel size^25^). The FDG uptake value of each voxel was normalized based on the whole-brain activity for each image^20^.

We first conducted voxel-based, one-way analysis of variance (ANOVA) on the data to compare the brain activities among the groups. After determining the cortical regions that corresponded to the main effect of the pain status (N, AG, and AP), we performed multiple-comparison analyses to localize the cortical regions demonstrating different activities between group pairings. The initial voxel threshold was *p* = 0.001 uncorrected, and regions were considered significant at a cluster family-wise error (FWE)-corrected threshold of *p* < 0.05 within the cerebral region. Since the prevalence of anxiolytic use was higher in the AP group, we also tested another ANOVA model with the same pain status factors and included anxiolytic use as a covariate. However, we could not include the anxiety severity in the current retrospective study owing to the lack of data.

Second, we investigated voxel-wise metabolic connectivity in the brains of patients from each group using interregional correlation analysis^26^. Metabolic connectivity determines regionally connected areas of the resting brain from static FDG-PET images collected from multiple participants. It determines the cortical connectivity with the strength of co-activation or deactivation between regions based on a conjugate increase or decrease in the cerebral metabolic rate of glucose^18,27^. We defined three seed regions obtained from the main effect analysis in the one-way ANOVA that differentiated the resting-state brain activity of the three groups. More specifically, the seed regions were defined as: (1) the anatomically parcellated region in the anatomical automatic labeling (AAL) atlas showing a significant main effect of enhanced glucose metabolism across groups, (2) volume exceeding 30 voxels, and (3) present in the cerebral cortex. The mean normalized FDG uptake values of these seed regions were used as independent variables in a general linear model to determine voxels that covaried with activity in the seed regions across patients. After obtaining the t-value map in the regression analysis, we extracted regions showing significant metabolic connectivity under the statistical criterion of FWE-corrected *p* values < 0.05 with a cluster-size threshold of k > 30.

Based on the reported engagement of pain status in the resting-state brain networks^28–35^, we investigated the metabolic connectivity from the seed regions occurring within the brain regions of the default mode network (DMN), salience network (SN), and central executive network (CEN). The triple network of DMN, SN, and CEN was investigated based on the causal relationship between the aberrant interactions within and between these networks and neuropsychiatric illnesses^32,36^. Cortical regions within the triple network were defined according to previous studies^28,37–39^. Brain masks representing each network were prepared using either the AAL3 atlas^40^ or Brodmann atlas implemented in the WFU PickAtlas^41^ (see Supplemental Table S1). The resultant activity map of metabolic connectivity was further masked with the mask image of each brain network for representation purposes. The detected regions were visualized on a single-subject T1 image using the xjView toolbox (https://www.alivelearn.net/xjview).

### Statistical analysis

We used one-way ANOVA or the chi-square test to examine differences in patient demographics among the groups. Multiple comparisons were corrected with the Scheffe test. Data were considered statistically significant at *p* < 0.05. Statistical analyses were performed using MedCalc version 19.8 (MedCalc Software, Ostend, Belgium).

## Results

### Clinical profiles

Age, sex, and cancer stage did not differ among the N, AG, and AP groups. The pain score (NRS) was significantly different between each group pairing (*p* < 0.001, Table 1). The number of cancer recurrences was higher in the AP group (*p* < 0.05). However, there was no statistically significant difference between the AG and the AP or N groups. The type of analgesic used showed a significant difference between the AG and AP groups (*p* < 0.001), while the strength of the opioids used and duration of opioid treatment were comparable. The number of patients who used anxiolytics was higher in the AP group (*p* < 0.05) while the usage rates were comparable between the AG and N groups.

### Differences in brain activities related to varying levels of perceived cancer pain and pain control

**Figure 1:**
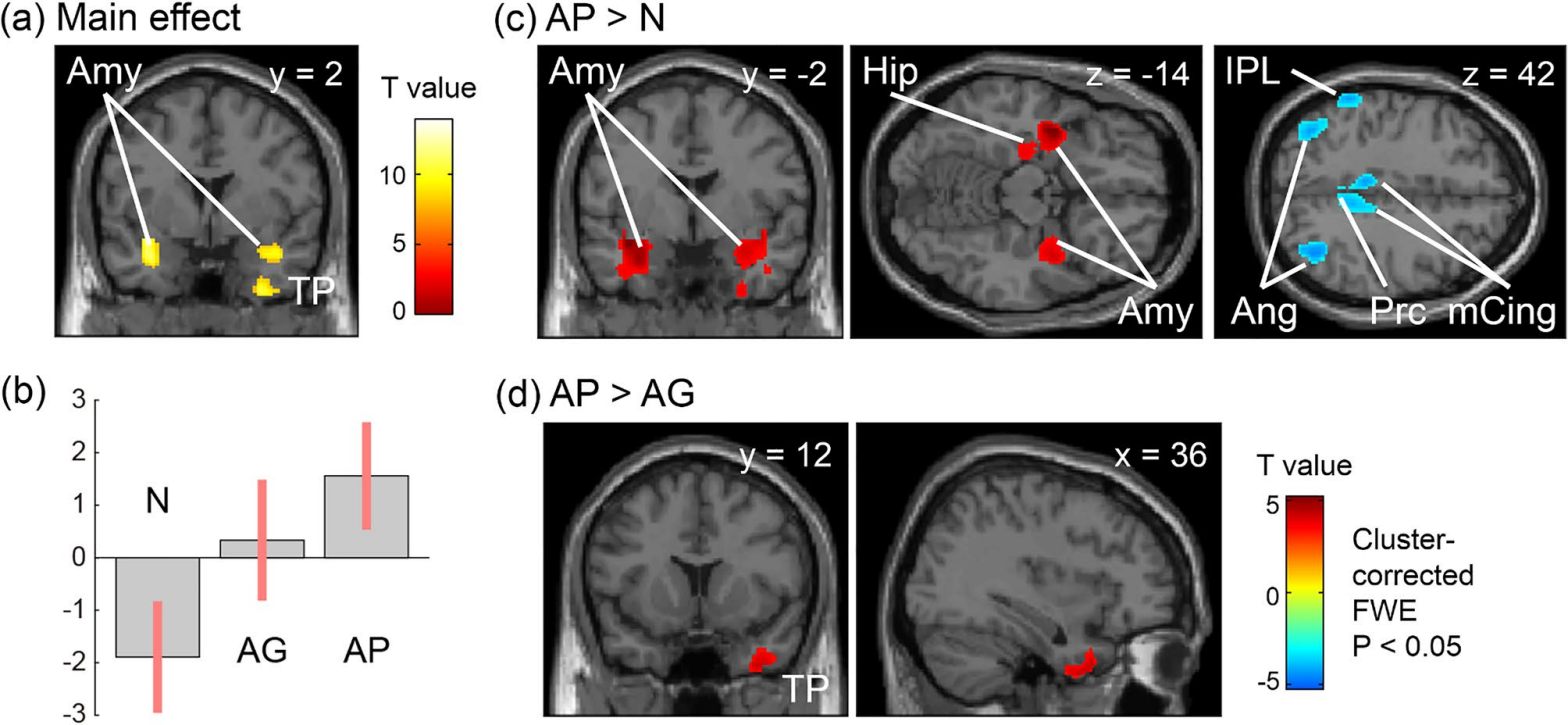
Differences in brain activities related to varying levels of perceived cancer pain (cluster corrected FWE, *p* < 0.05). A statistically significant main effect among the groups is shown in the bilateral amygdala (Amy) and right temporal pole (TP) (**a**). Representative contrast estimates and the 95% confidence interval in the left amygdala [− 26, − 2, − 20] demonstrated that glucose uptake increased in the order N, AG, and AP (**b**). Multiple comparisons further indicated enhanced activity within the amygdala and inhibited activity in the inferior parietal lobe, precuneus, and middle cingulate cortex in the AP group relative to the N group (**c**). The right TP shows increased activity in the AP group relative to the AG group (**d**). There are no significant activity differences between groups AG and N. *AG* patients with good pain control under analgesics; *Amy* amygdala, *Ang* angular gyrus, *AP* patients with poor pain control despite analgesic treatment, *Hip* hippocampus, *IPL* inferior parietal lobe, *mCing* middle cingulate gyrus, *N* patients not on analgesics, *Prc* precuneus, *TP* temporal pole.

One-way ANOVA showed the statistically significant main effect of enhanced glucose metabolism in the bilateral amygdala, right inferior temporal gyrus, and right temporal pole (TP) (Fig. 1a, b; see Supplemental Table S2) in the patient groups. Since the latter two regions were closely located and consist of continuous subclusters, they are concatenated as a single seed region and the subsequent three seed regions were selected for further metabolic connectivity analysis.

Multiple comparisons demonstrated significant differences in activity between the AP and other groups (Fig. 1c: AP > N included limbic regions, mainly the amygdala; AP < N included the inferior parietal lobe (IPL), temporoparietal junction, precuneus, and median cingulate; Fig. 1d: AP > AG included TP; see Supplemental Table S3). However, the cortical activity patterns were comparable between the N and AG groups.

The main effect in the left amygdala and right TP remained after anxiolytic use was included in the ANOVA model at the lower statistical threshold of uncorrected *p* < 0.001. Multiple comparisons also demonstrated increased bilateral amygdala and TP activity in the AP compared with the other groups (see Supplemental Fig. S1 and Supplemental Tables S4–S5; uncorrected *p* < 0.001).

### Metabolic connectivity focusing on brain networks

**Figure 2:**
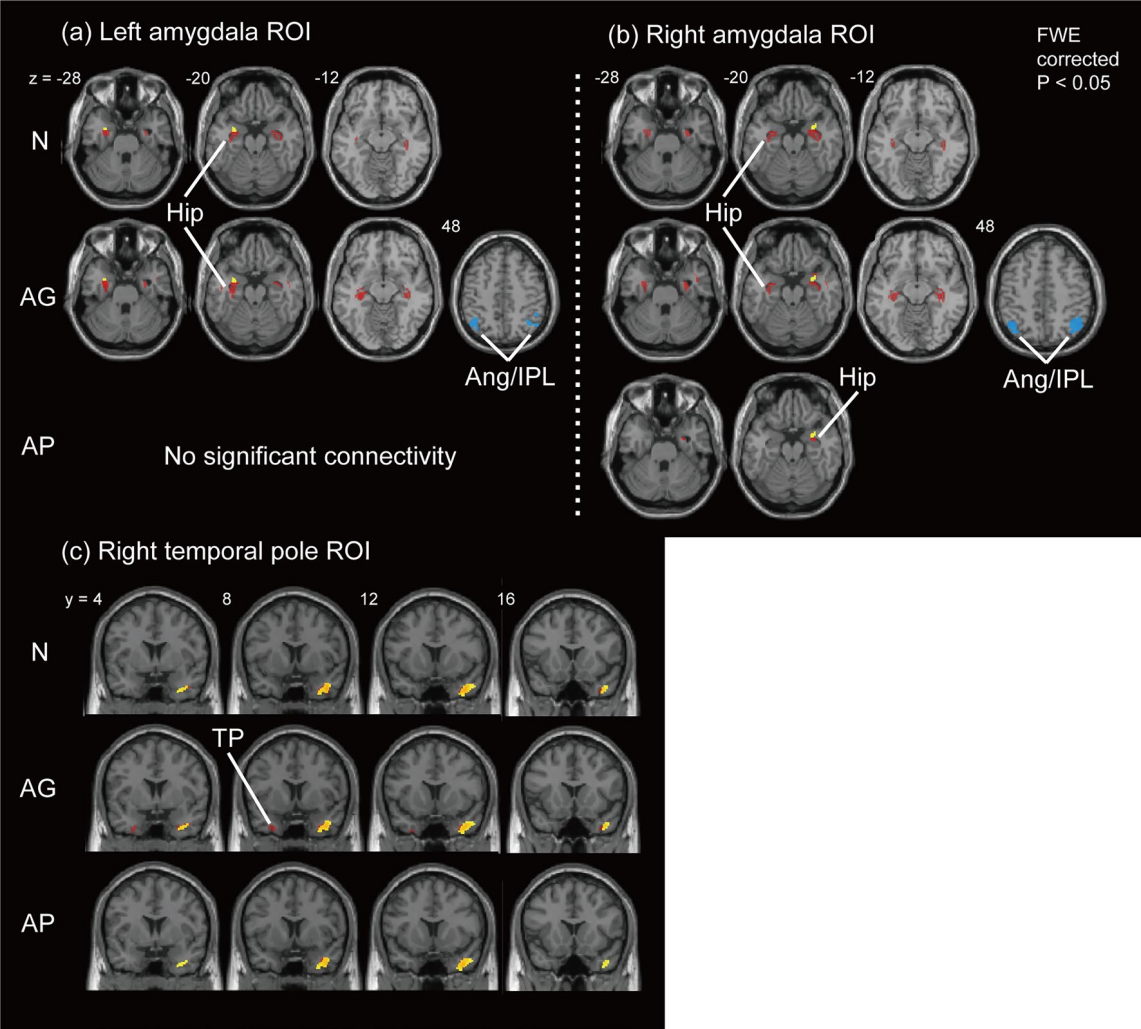
Brain regions showing metabolic connectivity from the left amygdala region of interest (ROI) (**a**), the right amygdala ROI (**b**), and the right temporal pole ROI (**c**) within the DMN regions. The seed ROIs are shown in yellow. Red and blue regions show the areas of co-activation and -deactivation with the seed ROIs (FWE corrected, *p* < 0.05), respectively, based on conjugate increase or decrease in the cerebral metabolic rate of glucose. *AG* patients with good pain control under analgesics, *Ang* angular gyrus, *AP* patients with poor pain control despite analgesic treatments, *FWE* family-wise error, *Hip* hippocampus, *IPL* inferior parietal lobe, *N* patients without analgesics, *TP* temporal pole.

Figure 2 shows the cortical regions of the DMN that revealed metabolic connectivity from the seed regions of the bilateral amygdala and right TP, resulted in group differences in FDG uptake in the main-effect ANOVA analysis. The AG and N groups showed almost the same metabolic connectivity pattern; bilateral hippocampal co-activity occurred with the amygdala seed regions within the DMN regions (Fig. 2a, b; see Supplemental Tables S6 and S7), while metabolic connectivity from the amygdala seed was mostly reduced in the AP group. Differences in metabolic connectivity patterns between the N and AG groups were characterized by additional connectivity in the posterior IPL (co-deactivation with amygdala seeds) in the AG group but not in the N group.

Metabolic connectivity from the TP seed showed mostly similar patterns of autocorrelation and correlation with neighboring regions within the TP regardless of patient group (Fig. 2c; see Supplemental Table S8). Additional correlated activity in the contralateral TP region was observed in the AG group.

**Figure 3:**
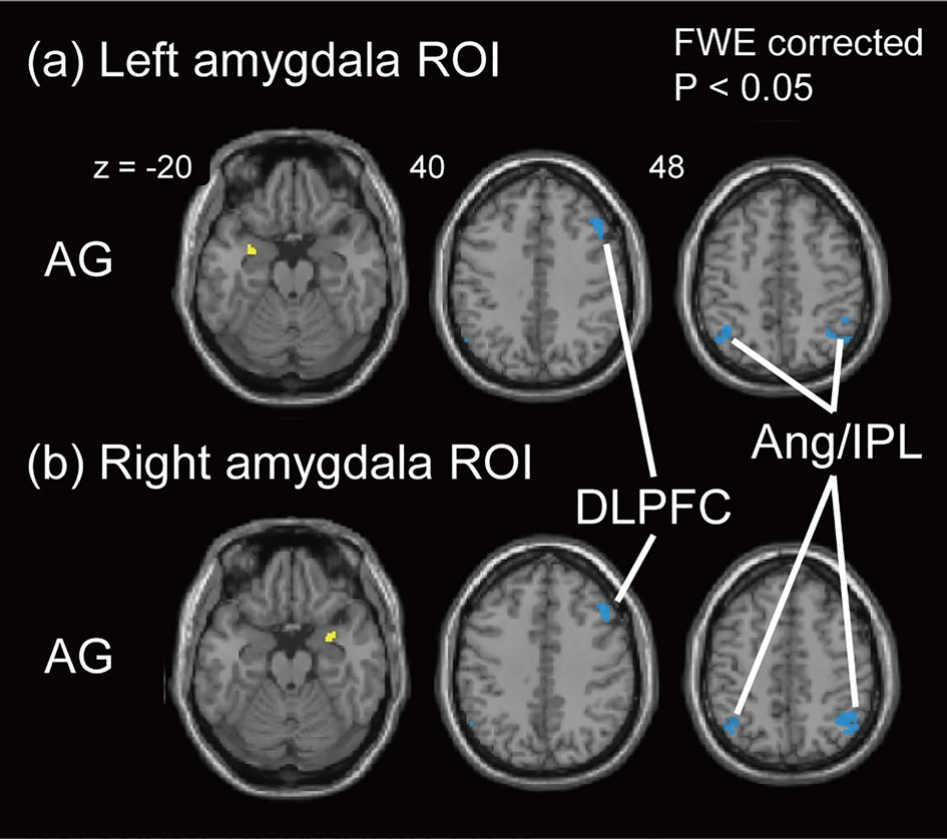
Brain regions showing metabolic connectivity from the left amygdala region of interest (ROI) (**a**) and the right amygdala ROI (**b**) within the CEN regions. The seed ROIs are shown in yellow. Statistically significant metabolic connectivity appeared only in the AG group from the bilateral amygdala ROIs. Only co-deactivation regions were found, which are shown in blue. *AG* patients with good pain control under analgesics, *Ang* angular gyrus, *CEN* central executive network, *DLPFC* dorsolateral prefrontal cortex, *FWE* family-wise error, *IPL* inferior parietal lobe.

Figure 3 shows the cortical regions of the CEN that demonstrated metabolic connectivity with the same seed regions. Significant co-deactivation with the bilateral amygdala seed regions was found in the right dorsolateral prefrontal cortex (DLPFC) and bilateral angular gyrus (Ang)/IPL regions within the CEN regions in the AG group (Fig. 3; see Supplemental Table S9). There was no significant metabolic connectivity in the other groups from any of the seed regions within the CEN. There was no significant metabolic connectivity within the SN in any of the groups. At a more liberal statistical threshold of uncorrected *p* < 0.001, the co-deactivation and co-activation with amygdala seed regions were observed within the CEN and SN regions, respectively, in all groups. The AG group showed more distributed co-deactivation within the CEN regions and co-activation of the insular cortex within the SN regions than that in the N and AP groups (see Supplemental Figs. S2–S4).

## Discussion

We retrospectively investigated the cortical representation of cancer pain using FDG-PET images. The resting-state glucose metabolism in the bilateral amygdala and right TP was greater in patients with poorly-controlled pain relative than that in patients with well-controlled pain and those without pain. Patients with poor pain control further demonstrated diminished co-activation between the amygdala and bilateral hippocampus, suggesting that the presence of alterations in the DMN are associated with chronic cancer pain.

The patient profiles were largely comparable across the groups in terms of age, sex, primary sites of cancer, and cancer stage. In addition to the perceived intensity of pain, significant differences across groups were found in the number of cancer recurrences and the use of anxiolytics. The AP group had a higher local recurrence rate and higher proportion of anxiolytic use. However, the recurrence rates of patients taking anxiolytics in the N, AG, and AP groups were 33.3%, 16.7%, and 61.5%, respectively (n, 1 vs. 1 vs. 8). Moreover, the recurrence rate of anxiolytic users was 45% (n, 10 vs. 12), indicating that the anxiolytic use and recurrence rates may not be associated. These results support the fact that pain is the most common symptom of cancer, especially when cancer recurs^42^. The increased use of anxiolytics in the AP group suggests that poorly controlled pain may not only be related to physical conditions, but also psychological factors, such as anxiety and depression. Clinically, anxiety and depression modulate pain perception^43,44^ and vice versa^45,46^. Consequently, patients with cancer commonly experience the physical symptoms of pain, as well as the affective symptoms of anxiety and depression^47^.

The enhanced activity found in the amygdala and hippocampus of patients with poorly controlled pain relative to those without pain could therefore represent the neural response to the emotional stress caused by cancer pain^48,49^, as well as to the physical pain itself^50–52^. Recent studies support the significant role of the amygdala and hippocampus in enhancing pain perception under anxiety^53^ and regulating anxiety^54–56^. Our results suggest that the altered resting-state activity of these medial temporal lobe regions may be caused by cancer pain and associated psychological stress.

The TP is involved in various cognitive and psychological processes^57–59^ including pain processing^60^. Functional connectivity analyses also support the involvement of the TP in modulating brain regions associated with the sensory-discriminative and affective-motivational aspects of pain, including the insula and amygdala, in patients with chronic pain^60^ and generalized anxiety disorders^61^. Our observation of enhanced TP activity in patients with uncontrollable cancer pain may thus imply maladaptation of the central circuitry of pain perception and increased anxiety.

Metabolic connectivity analysis further revealed hippocampal co-activation with the amygdala seed regions in the AG and N groups but not in the AP group. The hippocampus and parahippocampal gyrus are part of the medial temporal subsystem of the DMN, which is involved in self-inspection and monitoring of the internal environment^62^. The specifically determined metabolic connectivity observed between the amygdala and anterior hippocampus supports the previous functional magnetic resonance imaging-based functional connectivity reported in healthy participants^55^. The metabolic hypoconnectivity between the amygdala and hippocampus in the AP group suggests a maladaptive response to prolonged pain or stress-induced hyperalgesia^63,64^. The coactivation of the amygdala and hippocampus may suggest good control of pain or anxiety in patients with cancer pain.

The metabolic connectivity analysis also revealed significant co-deactivation with amygdala seed regions found in the bilateral angular gyrus within the DMN, bilateral IPL regions associated with the DMN and CEN, and right DLPFC within the CEN, which were observed exclusively in the AG group. Reflecting the essential roles of the angular gyrus, IPL, and DLPFC in the cognitive regulation of emotional responses^65^, our results suggest an antagonistic relationship between emotional responses in the amygdala and its regulatory responses in these frontoparietal regions in patients with good pain control^32^.

This study had several limitations. First, the psychological history, depression, and anxiety status of the patients were not available in this retrospective study. The perceived intensity of anxiety may varied among individuals^66^ therby affecting limbic system activity^67^. However, the included patients may have experienced similar emotional stress, as they had all been recently diagnosed with cancer or recurrence and underwent FDG-PET to determine the cancer stage. Furthermore, all three groups of patients used benzodiazepines for anxiety. Patients in the N and AP groups also took zolpidem, and one patient each took zopiclone, mirtazapine, quetiapine, and flupentixol/melitracen in the AP group. The reduced statistical significance of the perceived pain intensity-related activity in the medial temporal regions in patients taking controlled anxiolytics suggests a moderate effect of anxiety on the metabolic network in patients with severe cancer pain. However, the anxiety severity should be included to precisely investigate the effect of anxiety on the cortical activity related to cancer pain. Further prospective studies should incorporate information on anxiety and depression intensity to disentangle anxiety-related neural correlates from pain-related symptoms. Second, the type of analgesics was significantly different between the AG and AP groups. However, the effect of analgesics on cortical activity may have been minor because perceived pain intensity was not significantly different between users of weak and strong opioids in the AG and AP groups and the total durations of opioid use were comparable between the groups. However, various opioids may alter cerebral activity, which requires further investigation to identify the pharmacological mechanisms in cancer pain. Third, metabolic connectivity maps were only qualitatively compared since the metabolic connectivity analysis provided only a single connectivity map per group. Further studies should employ normal control data to determine the individual alternations in the metabolic connectivity architecture relative to control metabolic connectivity maps^68^ for between-group comparisons.

## Conclusions

PET imaging in patients with head and neck squamous cell carcinoma or esophageal cancer demonstrated the potential involvement of cancer pain in enhancing temporal lobe activity, especially in the amygdala, hippocampus, and TP regions. Since these regions are strongly involved in emotion and interoception, further studies should investigate the relationship between psychological conditions and pain in patients with cancer and analyze the resultant brain activity.

## Supplementary Information

Supplementary Information.

## Data Availability

The raw data are available on reasonable request from the corresponding author.
