# Supplementary material for: Altered metabolic connectivity between the amygdala and default mode network is related to pain perception in patients with cancer
Source: Sci Rep. 2022 Aug 18;12:14105. doi: 10.1038/s41598-022-18430-2 (PMC9388574; doi:10.1038/s41598-022-18430-2)
Supplement: Supplementary file 1 — Supplementary Information. [file 41598_2022_18430_MOESM1_ESM.pdf]

Supplementary Information for

Altered metabolic connectivity between the amygdala and default mode network is related to pain perception in patients with cancer

Wen-Ying Lin [1,2], Jen-Chuen Hsieh [3], Ching-Chu Lu [4], Yumie Ono [5*]
[1] Department of Anesthesiology, National Taiwan University Cancer Center, Taipei, Taiwan
[2] Department of Anesthesiology, National Taiwan University Hospital, Taipei, Taiwan
[3] Department of Biological Science and Technology, College of Biological Science and Technology, National Yang Ming Chiao Tung University, Taipei, Taiwan
[4] Department of Nuclear Medicine, National Taiwan University Hospital, Taipei, Taiwan
[5] School of Science and Technology, Meiji University, Kawasaki, Japan

***Corresponding author**: Yumie Ono, PhD
School of Science and Technology, Meiji University, 1-1-1 Higashi-Mita, Tama-ku, Kawasaki, Kanagawa 2148571, Japan
Tel: +81 44 934 7302, Fax: +81 44 934 7883, Email: yumie@meiji.ac.jp

This supplemental material consists of 9 supplemental tables (Table S1-S9) and 4 figures (Figure S1-S4). The anatomical location is described using AAL3 abbreviation (Rolls, E. T., Huang, C. C., Lin, C. P., Feng, J., & Joliot, M. (2020). Automated anatomical labelling atlas 3. *Neuroimage*, **206**, 116189) in Tables S2-S7.

Table S1 List of anatomical label numbers in AAL3 or Brodmann areas corresponding to brain network components.

Brain network and component regions	Anatomical labeling no. in AAL3	Brodmann area
**Default mode network**		
Anterior/dorsal/posterior medial prefrontal cortex	19-24	
Posterior cingulate cortex	39,40	
Temporal pole	87, 88, 91, 92	
Lateral temporal cortex	89, 90	
Temporoparietal junction	67-70, 85, 86	
Posterior inferior parietal lobule	65, 66	
Hippocampal formation	41, 42	
Parahippocampal cortex	43, 44	
Restosplenial cortex		26, 29, 30
**Salience network**		
Anterior/posterior insula	33, 34	
Dorsal anterior cingulate cortex	151-156	
Hypothalamus	NA	
Periaqueductal gray	NA	
VStr, ventral striatum	17, 18, 157, 158	
VTA, ventral tegmental area	159, 160	
**Central executive network**		
Dorsolateral prefrontal cortex		9, 46
Lateral parietal area		39, 40

NA: none available.

Table S2. Cluster information of the main effect of ANOVA analysis in groups N, AG, and AP.

Cluster p value (FWE corrected)	Cluster volume (k_E)	Center of gravity MNI coordinates [mm]			Anatomical location
		x	y	z	
**0.021**	**244**	**-33**	**-2**	**-19**	
	48				Amygdala_L
	15				Insula_L
	15				Temporal_Pole_Sup_L
	12				Hippocampus_L
**0.001**	**451**	**38**	**6**	**-33**	
	206				Temporal_Pole_Mid_R
	87				Temporal_Inf_R
	31				Amydgala_R
	22				Fusiform_R
	21				Temporal_Pole_Sup_R
**0.000**	**529**	**5**	**-23**	**-33**	
	529				Pons

The coordinates of the center of gravity of each cluster and their total volumes were provided in bold fonts. Activated volumes of anatomically separated regions within the same cluster were also provided.

Table S3. Cluster information of the post-hoc multiple comparison among groups N, AG, and AP.

Cluster p value (FWE corrected)	Cluster volume (k_E)	Center of gravity MNI coordinates [mm]			Anatomical location
		x	y	z	
AP > N					
**< 0.001**	**996**	**-33**	**-2**	**-26**	
	181				Temporal_Pole_Inf_L
	164				Hippocampus_L
	81				Amygdala_L
	63				Temporal_Pole_Mid_L
	59				Fusiform_L
	40				Temporal_Mid_L
	32				ParaHippocampal_L
	25				Temporal_Sup_L
	22				Temporal_Pole_Sup_L
	22				Insula_L
**< 0.001**	**951**	**38**	**3**	**-29**	
	281				Temporal_Pole_Mid_R
	102				Temporal_Inf_R
	96				Amygdala_R
	73				Fusiform_R
	55				Temporal_Pole_Sup_R
	28				Insula_R
	25				Amygdala
	22				Temporal_Mid_R
	19				Hippocampus_R
	11				ParaHippocampal_R
N > AP					
**0.006**	**436**	**41**	**-67**	**37**	
	309				Angular_R
	110				Occipital_Mid_R
	15				Occipital_Sup_R

Table S3. (continued)

	**0.025**	**310**	**-39**	**-65**	**43**	
		182				Angular_L
		95				Parietal_Inf_L
		33				Occipital_Mid_L
	**0.006**	**432**	**1**	**-36**	**40**	
		200				Cingulum_Mid_L
		124				Cingulate_Mid_R
		73				Precuneus_R
		28				Precuneus_L
	**0.010**	**386**	**-57**	**-47**	**34**	
		144				SupraMarginal_L
		135				Parietal_Inf_L
		79				Angular_L
AP > AG						
	**0.045**	**262**	**39**	**6**	**-40**	
		135				Temporal_Inf_R
		114				Temporal_Pole_Mid_R

The coordinates of the center of gravity of each cluster and their total volumes were provided in bold fonts. Activated volumes of anatomically separated regions within the same cluster were also provided.

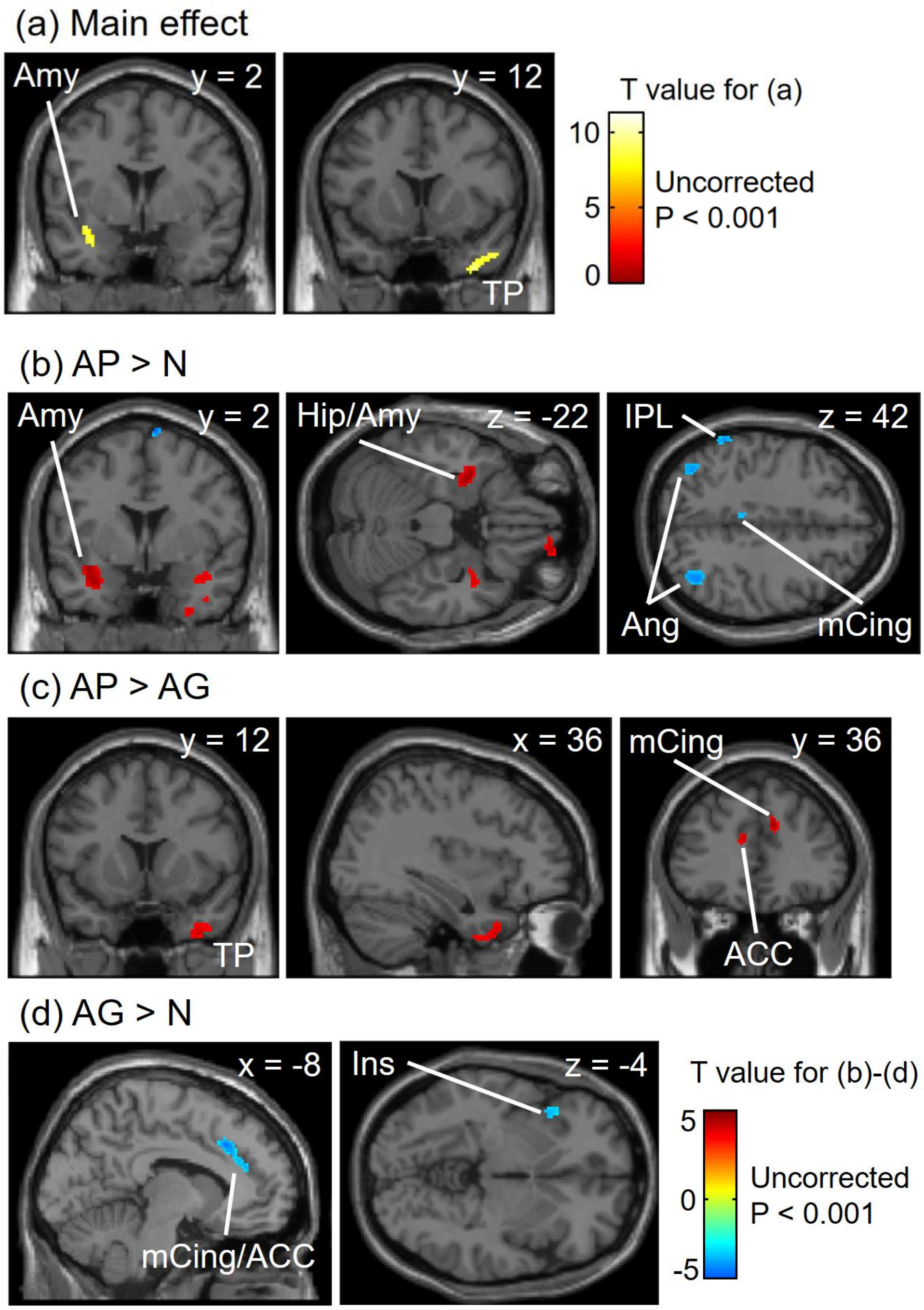

Figure S1. Differences in brain activities related to varying levels of perceived cancer pain with anxiolytics use as a covariate (uncorrected, $p < 0.001$). A statistically significant main effect among the groups is shown in the left amygdala regions and right temporal pole (TP) (**a**). Multiple comparisons

indicated enhanced activity within the amygdala, insula, hippocampus, and temporal pole in the AP group relative to the N group. Inhibited activity was found in the angular gyrus, inferior parietal lobe, posterior part of middle cingulate cortex, and precuneus (**b**). The right TP, anterior middle cingulate cortex, and anterior cingulate cortex showed increased activity in the AP group relative to the AG group (**c**). There is significantly inhibited activity in the anterior part of middle cingulate cortex, anterior cingulate cortex, and insular in the AG group relative to the N group (**d**). N: patients not on analgesics, AG: patients with good pain control under analgesics, AP: patients with poor pain control despite analgesic treatment. ACC: Anterior cingulate cortex, Amy: amygdala; Ang: angular gyrus; Hip: hippocampus; Ins: insular; IPL: inferior parietal lobe; mCing: middle cingulate gyrus; Prc: precuneus; TP: temporal pole.

Table S4. Cluster information of the main effect of ANOVA analysis with anxiolytics use as covariate in groups N, AG, and AP.

Peak p value (uncorrected)	Cluster volume (k_E)	MNI coordinates [mm]			Anatomical location
		x	y	z	
**< 0.001**	**92**	**-35**	**-1**	**-18**	
	13				Amygdala_L
	12				Insula_L
**< 0.001**	**166**	**2**	**-18**	**-32**	
	166				Pons
**< 0.001**	**87**	**39**	**12**	**-38**	
	72				Temporal_Pole_Mid_R
	14				Temporal_Inf_R

The coordinates of the center of gravity of each cluster and their total volumes were provided in bold fonts. Activated volumes of anatomically separated regions within the same cluster were also provided.

Table S5. Cluster information of the post-hoc multiple comparison with anxiolytics use as covariate among groups N, AG, and AP.

Peak p value (uncorrected)	Cluster volume (k_E)	MNI coordinates [mm] x	y	z	Anatomical location
AP > N					
**< 0.001**	**272**	**-34**	**-2**	**-19**	
	49				Amygdala_L
	19				Temporal_Sup_L
	19				Insula_L
	15				Hippocampus_L
	12				Temporal_Pole_Sup_L
**< 0.001**	**603**	**3**	**-21**	**-33**	
	603				Pons
**< 0.001**	**242**	**39**	**9**	**-37**	
	175				Temporal_Pole_Mid_R
	42				Temporal_Inf_R
	24				Fusiform_R
**< 0.001**	**40**	**40**	**3**	**-22**	
	11				Temporal_Pole_Sup_R
N > AP					
**< 0.001**	**138**	**13**	**-64**	**30**	
	119				Precuneus_R
	19				Cuneus_R
**< 0.001**	**308**	**42**	**-67**	**36**	
	221				Angular_R
	80				Occipital_Mid_R
**< 0.001**	**138**	**-39**	**-68**	**38**	
	90				Angular_L
	41				Occipital_Mid_L
**< 0.001**	**139**	**-59**	**-45**	**35**	
	55				SupraMarginal_L
	51				Parietal_Inf_L

Table S5. (continued)

		17				Angular_L
	**< 0.001**	**33**	**-6**	**-31**	**39**	
		33				Cingulate_Mid_L
AP > AG						
	**< 0.001**	**51**	**12**	**35**	**31**	
		31				Cingulate_Mid_R
	**< 0.001**	**38**	**-9**	**39**	**20**	
		17				ACC_pre_L
		13				Frontal_Sup_Medial_L
	**< 0.001**	**141**	**38**	**8**	**-41**	
		72				Temporal_Pole_Mid_R
		67				Temporal_Inf_R
	**< 0.001**	**34**	**-43**	**-11**	**-34**	
		34				Temporal_Inf_L
N > AG						
	**< 0.001**	**70**	**-64**	**-37**	**-16**	
		42				Temporal_Inf_L
		28				Temporal_Mid_L
	**< 0.001**	**71**	**-7**	**29**	**34**	
		39				Frontal_Sup_Medial_L
		15				Cingulate_Mid_L
		10				ACC_sup_L
	**< 0.001**	**52**	**-48**	**37**	**16**	
		35				Frontal_Inf_Tri_L
		17				Frontal_Mid_2_L
	**< 0.001**	**35**	**23**	**38**	**-20**	
		20				OFCant_R
		15				OFCmed_R
	**< 0.001**	**40**	**-42**	**19**	**-3**	
		19				Insula_L
		11				Frontal_Inf_Tri_L

The coordinates of the center of gravity of each cluster and their total volumes were provided in bold fonts. Activated volumes of anatomically separated regions within the same cluster were also provided.

Table S6. Metabolic connectivity results from seed region of left amygdala ROI within DMN regions.

P value (FWE corrected)	Cluster volume (k_E)	MNI coordinates [mm] x	y	z	Anatomical location
**group N**					
Co-activation					
< 0.001	163	-32	-4	-26	Hippocampus_L
< 0.001	216	38	-16	-18	Hippocampus_R
< 0.001		34	-4	-24	Hippocampus_R
0.002		36	4	-32	Temporal_Pole_Mid_R
**group AG**					
Co-activation					
< 0.001	413	-30	-6	-20	Hippocampus_L
< 0.001		-30	0	-30	ParaHippocampal_L
< 0.001		-30	-20	-14	Hippocampus_L
< 0.001	47	-46	-24	-18	Temporal_Mid_L
< 0.001		-46	-14	-18	Temporal_Mid_L
< 0.001	228	34	-4	-22	Hippocampus_R
< 0.001		30	0	-30	ParaHippocampal_R
0.001		36	-24	-10	Hippocampus_R
< 0.001	44	50	-8	-22	Temporal_Mid_R
0.005		46	-2	-26	Temporal_Mid_R
0.012		44	-6	-14	Temporal_Sup_R
Co-deactivation					
0.007	119	-42	-60	50	Parietal_Inf_L
0.013		-38	-54	46	Parietal_Inf_L
0.023		-52	-62	42	Parietal_Inf_L
0.012	59	38	-54	46	Parietal_Inf_R
0.019		52	-62	48	Angular_R
0.016	35	52	-48	50	Parietal_Inf_R

Table S7. Metabolic connectivity results from seed region of right amygdala ROI within DMN regions.

P value (FWE corrected)	Cluster volume (k_E)	MNI coordinates [mm] x	y	z	Anatomical location
**group N**					
Co-activation					
< 0.001	351	32	-4	-22	Hippocampus_R
< 0.001		36	4	-32	Temporal_Pole_Mid_R
0.002		38	-22	-14	Hippocampus_R
< 0.001	204	-26	-6	-20	Hippocampus_L
0.001		-32	-4	-26	Hippocampus_L
**group AG**					
Co-activation					
< 0.001	504	32	-4	-22	ParaHippocampal_R
< 0.001		36	4	-32	Temporal_Pole_Mid_R
< 0.001		36	-24	-10	Hippocampus_R
< 0.001	35	34	4	-22	Temporal_Pole_Sup_R
< 0.001	321	-32	-4	-26	Hippocampus_L
< 0.001		-28	2	-34	Temporal_Pole_Sup_L
< 0.001		-30	-22	-12	Hippocampus_L
Co-deactivation					
0.001	218	40	-76	40	Angular_R
0.001		40	-62	48	Angular_R
0.015		52	-62	46	Angular_R
0.001	112	-36	-68	48	Parietal_Inf_L
0.032		-54	-58	42	Parietal_Inf_L
**group AP**					
Co-activation					
< 0.001	80	32	-4	-22	Hippocampus_R
< 0.001		28	0	-30	ParaHippocampal_R

Table S8. Metabolic connectivity results from seed region of right temporal pole ROI within DMN regions.

P value (FWE corrected)	Cluster volume (k_E)	MNI coordinates [mm] x	y	z	Anatomical location
**group N**					
Co-activation					
< 0.001	203	40	10	-36	Temporal_Pole_Mid_R
**group AG**					
Co-activation					
< 0.001	228	38	10	-36	Temporal_Pole_Mid_R
< 0.001	66	-30	4	-38	Temporal_Pole_Mid_L
**group AP**					
Co-activation					
< 0.001	151	40	10	-36	Temporal_Pole_Mid_R

Table S9. Metabolic connectivity results from seed region of amygdala ROIs within CEN regions.

P value (FWE corrected)	Cluster volume (k_E)	MNI coordinates [mm] x	y	z	Anatomical location
**group AG, left amygdala ROI**					
Co-deactivation					
< 0.001	75	44	28	40	Frontal_Mid_R
0.010		48	22	34	Frontal_Mid_R
0.007	92	-42	-60	50	Parietal_Inf_L
0.013		-38	-54	46	Parietal_Inf_L
0.019		-54	-62	42	Parietal_Inf_L
0.012	46	38	-54	46	Parietal_Inf_R
0.019		52	-62	48	Angular_R
0.016	34	52	-48	50	Parietal_Inf_R
**group AG, right amygdala ROI**					
Co-deactivation					
< 0.001	72	44	28	40	Frontal_Mid_R
0.020		48	22	34	Frontal_Mid_R
0.001	122	40	-60	48	Angular_R
0.015		52	-62	46	Angular_R
0.005	65	-40	-64	48	Angular_L
0.026		-54	-60	42	Parietal_Inf_L

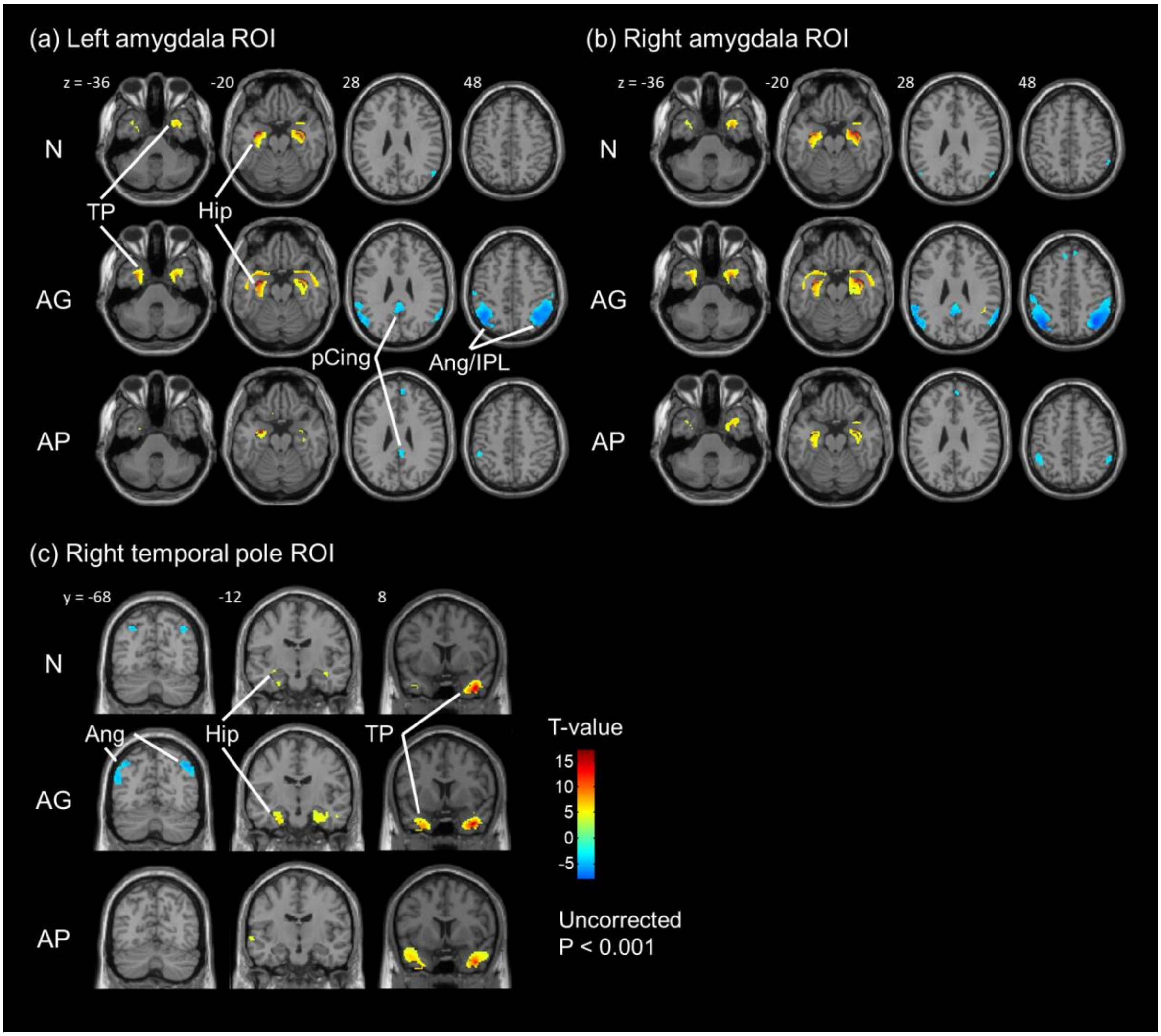

Figure S2. Brain regions showing metabolic connectivity from the left amygdala region of interest (ROI) (**a**), the right amygdala ROI (**b**), and the right temporal pole ROI (**c**) within the DMN regions for liberal statistical significance (uncorrected $p < 0.001$). The seed ROI was not present for visibility of t-value distribution but was identical as in Figure 2. Positive and negative t-values show the areas of co-activation and -deactivation with the seed ROIs, respectively, based on conjugate increase or decrease in the cerebral metabolic rate of glucose. The AG and N groups showed almost the same co-activation pattern of bilateral

hippocampus occurred with the amygdala seed regions within the DMN regions, while metabolic connectivity from the amygdala seed was mostly reduced in the AP group. The co-deactivation with amygdala seeds are found in the angular gyrus and inferior parietal lobes in the AG group. The posterior cingulate co-deactivation with the amygdala seeds was found in the AG and AP groups. Metabolic connectivity from the TP seed showed mostly similar patterns of autocorrelation and correlation with neighboring regions within the TP regardless of patient group. Co-activation in the contralateral TP region was observed in all groups but the correlated region was larger in the AG and AP groups. The bilateral hippocampus co-activation and the angular gyri co-deactivation were found in the N and AP groups, while the active areas were larger in the AG group. N: patients without analgesics, AG: patients with good pain control under analgesics, AP: patients with poor pain control despite analgesic treatments. Ang: angular gyrus; Hip: hippocampus; IPL: inferior parietal lobe; pCing: post cingulate gyrus; TP: temporal pole.

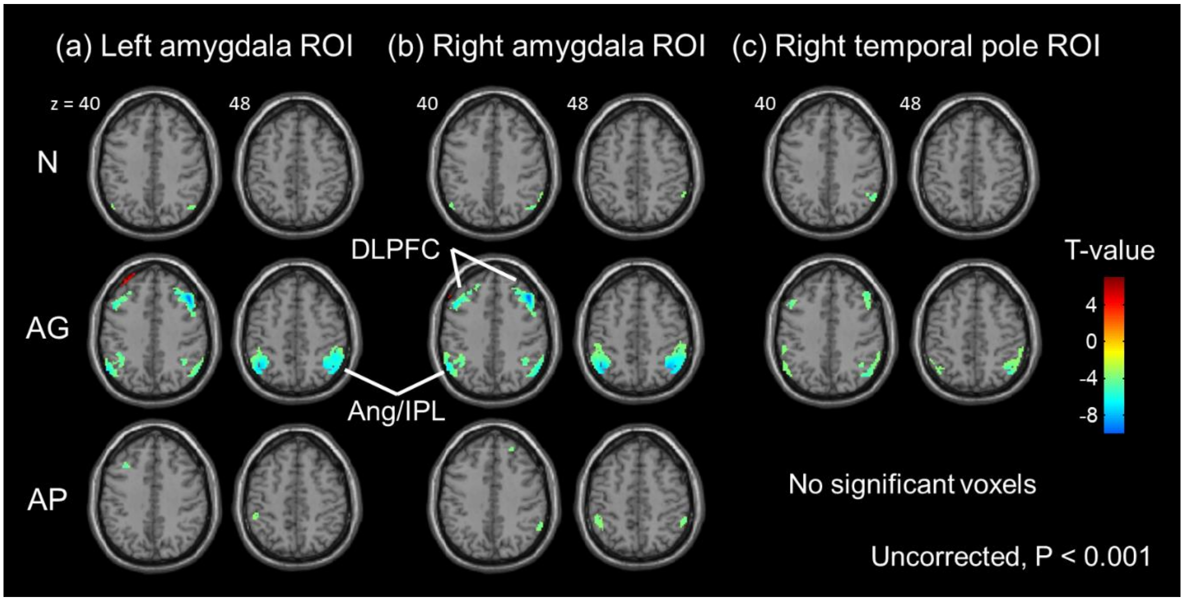

Figure S3. Brain regions showing metabolic connectivity from the left amygdala region of interest (ROI) (**a**), the right amygdala ROI (**b**), and the right temporal pole ROI (**c**) within the CEN regions for liberal statistical significance (uncorrected $p < 0.001$). The seed ROI was not present for visibility of t-value distribution but was identical as in Figure 2. Positive and negative t-values show the areas of co-activation and -deactivation with the seed ROIs, respectively, based on conjugate increase or decrease in the cerebral metabolic rate of glucose. Significant co-deactivation with the bilateral amygdala seed regions was found in the bilateral angular gyrus (Ang)/IPL regions within the CEN regions in all groups, however, the active regions were larger in the AG group. The AG group showed additional co-deactivation in the dorsolateral prefrontal cortex with the bilateral amygdala and the temporal pole seed regions. N: patients without analgesics, AG: patients with good pain control under analgesics, AP: patients with poor pain control

despite analgesic treatments. Ang: angular gyrus; DLPFC: dorsolateral prefrontal cortex; IPL: inferior parietal lobe.

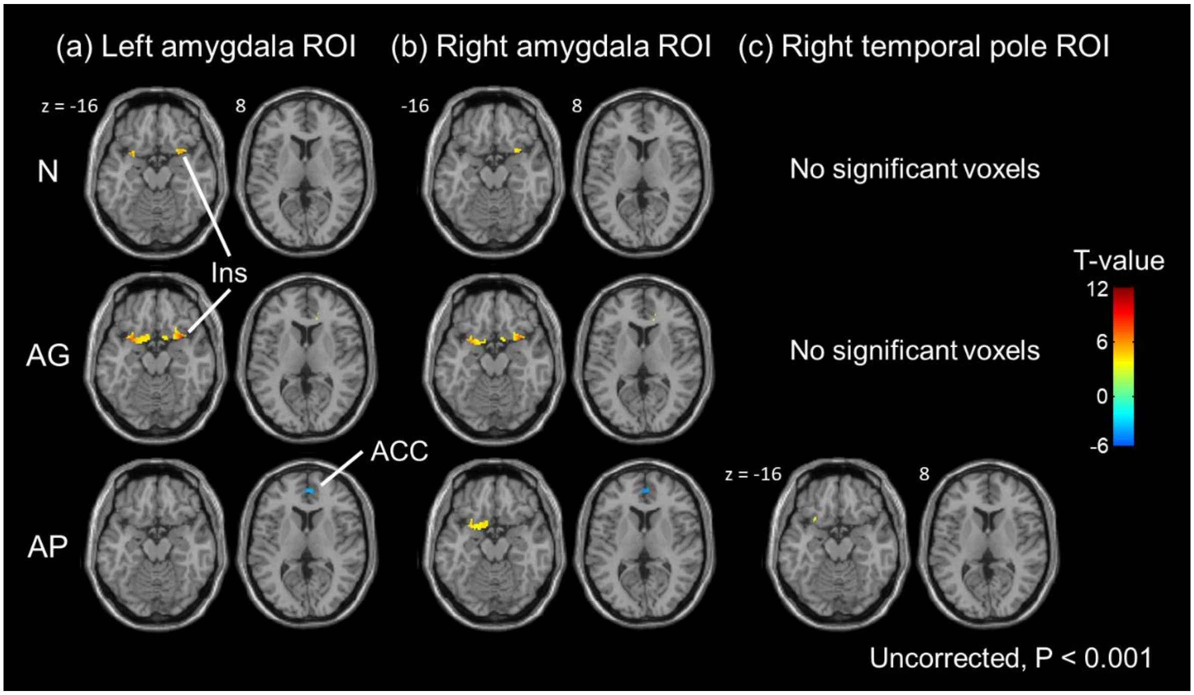

Figure S4. Brain regions showing metabolic connectivity from the left amygdala region of interest (ROI) (**a**), the right amygdala ROI (**b**), and the right temporal pole ROI (**c**) within the SN regions for liberal statistical significance (uncorrected $p < 0.001$). The seed ROI was not present for visibility of t-value distribution but was identical as in Figure 2. Positive and negative t-values show the areas of co-activation and -deactivation with the seed ROIs, respectively, based on conjugate increase or decrease in the cerebral metabolic rate of glucose. Significant co-activation with the bilateral amygdala seed regions was found in the bilateral insular regions within the SN regions in the N and AG groups. Significant co-deactivation with the bilateral amygdala seed regions was found in the anterior cingulate cortex within the SN regions in the AP group. N: patients without analgesics, AG: patients with good pain control under analgesics, AP:

patients with poor pain control despite analgesic treatments. ACC: Anterior cingulate cortex, Ins: insular.
